# Supplementary material for: Neutral vs. non-neutral genetic footprints of Plasmodium falciparum multiclonal infections
Source: PLoS Comput Biol. 2023 Jan 3;19(1):e1010816. doi: 10.1371/journal.pcbi.1010816 (PMC9838855; doi:10.1371/journal.pcbi.1010816)
Supplement: Attachment — Submitted filename: PCOMPBIOL-D-22-01024_review.pdf [file pcbi.1010816.s022.pdf]

The authors performed simulations to study the accuracy of two methods used to estimate MOI, THE REAL McCOIL and varcoding. I think this study addresses an important question and the results can provide insights into how to choose the most appropriate approach to estimate MOI. Since the simulations in THE REAL McCOIL paper did not show underestimation or overestimation of MOI, this manuscript can be improved by explaining this discrepancy.

1. The original THE REAL McCOIL paper presented simulation results that suggest unbiased estimates of MOI, so the readers may wonder what leads to different conclusions between studies. Is this due to the intrinsic differences between the two simulation frameworks? Can the authors provide any intuitive explanation or discuss this a bit more? I think the following analysis may help provide some hints:
   (a) Instead of using THE REAL McCOIL to estimate allele frequencies, take the true MAF from the simulations as input, use COIL (Galinsky et al. 2015, Malaria Journal) to estimate MOI, and see if MOI is still over or underestimated. This will help understand whether over or underestimated MOI is due to inaccurate MAF estimates. Line 290-296 mentioned that the less accurate MOIs were explained by highly inaccurate estimated MAF - it would be helpful to explore this more systematically.
   (b) After controlling for MAF (using the true MAF), if MOI is still over or underestimated, it is likely because the frequency of DAC is different from that expected in the model implemented in THE REAL McCOIL (Equation 2). If the proportion of DAC is higher (or lower) than expected from the REAL McCOIL model, MOI is likely to be overestimated (or underestimated). If this is the case, it would be helpful if the authors could provide some explanation for this (for example, can diversifying selection or immune selection and linkage between neutral and non-neutral markers explain why individuals with high MOI show lower than expected diversity and individuals with low MOI show higher than expected diversity)?

2. Line 238-239, "Interestingly, while most hosts with MOI < 3 show accurate MOI estimates, some can differ from the true MOI by up to 18, 12, and 13 co-infections for the low-, moderate-, and high-transmission simulations, respectively." Do these individuals show unusually high proportions of DAC?

3. The 95% credible interval of MOI from THE REAL McCOIL may be wider for higher values of true MOI. Therefore, I suggest also using "whether the 95% credible interval of MOI contains true MOI" to assess the accuracy of MOI estimates.

Minor comments

1. Does Fig 3 present values from simulations for high, moderate, or low transmission setting? I noticed that the proportion of DAC is 0 for true MOI = 1, but in Fig 1A, the estimated MOI can be greater than one low transmission setting, which is confusing.

2. Line 586-588 "We performed the categorical method of THE REAL McCOIL with a minor allele frequency (MAF) of 10% for a SNP to be considered." Why were only SNPs with MAF >10% considered? Are the results similar when another threshold (such as 5% or

1%) is used? For example, line 281-284, “Consequently, due to the high number of SNP loci, and thus individuals, that could not be considered in THE REAL McCOIL analysis, MOI could not have been estimated for any of the low-transmission setting simulations when a subsampling of 200 individuals was applied (S6 Fig).” Can MOI be estimated if the threshold is lowered?

3. How “distinct initial SNP allele frequencies” are set (were these frequencies sampled from a particular distribution)? It seems that it makes more sense to consider various allele frequencies for the default setting (compared to a constant frequency). Also, I only saw a range for the constant frequency in Table S1, but could not find what value was used.

4. If I understand it correctly, the true MOI defined in this study is the number of co-infections, no matter whether the parasites in different infections are genetically similar or not. However, in the abstract, MOI is defined as the number of genetically distinct parasite strains, and how to define “genetically distinct” is usually unclear and can lead to confusion. It would be helpful to explain/define these more clearly in the manuscript. For example, if an individual is infected twice and the parasites are 50% similar, is true MOI 2 or 1.5?

5. Line 344-346, “Consistent with the average PTS, the similarity networks for simulations under **high**-transmission settings did not group the var repertoires into well-defined modules while the similarity networks for simulations under **high**-transmission settings did.” There seems to be a typo here; one of them should be low- and moderate-.

6. Line 435-437, “Surprisingly, although THE REAL McCOIL approach assumes that genotyped SNP loci do not exhibit significant LD, the simulations performed with linked SNP loci did not show less reliable MOI estimation.” The same finding was also reported in the original paper.

7. Line 591-593, “… a probability of calling single allele loci double allele loci and of calling double allele loci single allele loci of 0.05 which were estimated with MOI and the allele frequencies) [21,66]”. I suggest adding “initial” to make it clearer. “… **an initial** probability of calling single allele loci double allele loci and of calling double allele loci single allele loci of 0.05 which were estimated with MOI and the allele frequencies) [21,66]”.
