# Supplementary material for: Neutral vs. non-neutral genetic footprints of Plasmodium falciparum multiclonal infections
Source: PLoS Comput Biol. 2023 Jan 3;19(1):e1010816. doi: 10.1371/journal.pcbi.1010816 (PMC9838855; doi:10.1371/journal.pcbi.1010816)
Supplement: Attachment — Submitted filename: Labbe_2022_ResponseToReferees_F.pdf [file pcbi.1010816.s023.pdf]

Neutral vs. non-neutral genetic footprints of

***Plasmodium falciparum* multiclonal infections**

Responses to editor, followed by those to referees.
(All the comments are in bold).

The authors present an interesting and relevant assessment of two methods for estimating the multiplicity of infection from population level parasite genetic data. This work essentially involves using an existing agent based model of malaria transmission, with extensions to monitor genetic features of the transmission, and applying the two procedures to the simulated data. The essential conclusions are that both methods have various levels of success and failure at different transmission intensities, but the authors better agreement of the mean MOIs estimated using the *var*coding approach (compared with the true MOIs) than THE REAL McCOIL approach:

- **True mean MOIs: 1.08 ± 0.30, 1.61 ± 0.95, and 3.84 ± 3.31**
- **THE REAL McCOIL: 1.27 ± 1.60, 1.93 ± 1.93, and 4.80 ± 4.60**
- ***Var*coding: 1.08 ± 0.29; 1.60 ± 0.93; and 3.74 ± 3.14**

While this study appears interesting and the results sensibly analysed, the major conclusions of the study may be highly dependent on the particular transmission model used to simulate the data, and in particular, the mechanisms of genetic diversity included in the model, as well as the source of multiple infections. This is outlined by both reviewers and these concerns must be addressed by the authors before the manuscript can be considered for publication in PLOS Computational Biology. Similar to both of the reviewer's comments on dissecting why there is agreement or lack of agreement between the simulated data and the two MOI approaches, the authors should consider:

(1) since the model appears to assume infection duration is determined solely by antigen variation and immune escape by *var* genes, the model should consider the possibility that infections also resolve randomly before exhaustion of the *var* gene repertoire (for example

due to other mechanisms of infection control) - and whether this impacts on the performance of the MOI estimation.

We have extended the model and sampling of the simulations in the following ways as recommended by referee 1: (1) we implemented in the model a way to randomly clear some infections, (2) we sampled infections throughout the season rather than at a point in time during that season, and (3) we sampled infections during transient rather than stationary dynamics, after simulating a temporary intervention reducing transmission.

For (1), the extended model implementation introduces a random clearance (with parameter "background_clearance_rate", S1 Table) to represent clearance processes not explicitly represented (for example, from infection control). We explored how the random clearance of infection impacts the performance of the MOI estimation methods by performing simulations with 48 unlinked SNP loci with homogenous initial allelic frequencies for the three transmission settings (S1 and S2 Tables). This design results in three distinct combinations of parameters and we ran 10 replicates per combination. In comparison to simulations without background clearance, the simulations with background clearance showed a reduction in the accuracy of the MOI estimated with THE REAL McCOIL approach for the three transmission settings (S9 and S11 Figs). This was especially observed for low true MOI values (i.e., from 1 to 3). However, no change in accuracy was observed between simulations generated with or without background clearance when the *var*coding approach was used (S9 and S11 Figs). These results suggest that the *var*coding approach allows robust estimation despite infection clearance due to processes other than the specific immunity conferred by the memory of the variant surface antigens encoded by *var* genes, such as generalized immunity. These new results are illustrated (S9 and S11 Figs), described (L306-308, and L375-377, and L713-720), and discussed (L462-466) in the revised version of the manuscript.

(2) It is a little unclear but it seems the model assumes that sexual reproduction can occur but the source of donor parasites is always from a single host? If the model includes the possibility that multiple hosts can infect a single mosquito, how will this impact the diversity and performance of the methods?

Indeed, during the sexual reproduction our model assumes that the source of the donor parasites is always a single host. We now provide a better explanation of how the transmission event and associated recombination are implemented (L613-630).

Although it is possible for a mosquito to bite several hosts within a short period of time so that the gametes from different hosts are of similar developmental stage to form zygotes, such bites are much less likely than those transmitting infection from a single host. The average number of bites one falciparum mosquito takes in its whole lifetime (~20 days) is estimated to be around 3.88 in high endemic regions and 1.24 in low transmission regions (Garrett-Jones C, Shidrawi GR. Malaria vectorial capacity of a population of *Anopheles gambiae*: an exercise in epidemiological entomology. Bulletin of the World Health Organization. 1969;40: 531). In addition, gravid mosquitoes usually rest and lay eggs in between blood-meals, which are spaced 2-3 days apart. Given these biting behaviors, we did not model the possibility that multiple hosts infect a single mosquito.

Moreover, as genetic diversity and transmission intensity are highly interconnected, we do not expect a change to our implementation, allowing for multiple donors to a single recipient, to change the results for high-transmission settings given that overlap between *var* repertoires is extremely limited.

Finally, as interrupted feeds, where a single genotype from the same mosquito is inoculated into two people, can occur but not frequently, we also do not expect consideration of this process to change our results for low- and moderate-transmission settings (Conway DJ, McBride JS. Genetic evidence for the importance of interrupted feeding by mosquitoes in the transmission of malaria. Transactions of the Royal Society of Tropical Medicine and Hygiene. 1991;85: 454–456. doi:10.1016/0035-9203(91)90217-M). These points are discussed in the revised version of the manuscript (L532-539).

The editor suggests that the reviewers comments can be most adequately addressed by stress testing the structural assumptions of the model used for simulation (as outlined by Reviewer 1 and above), and/or by adapting a completely unique model of malaria

transmission from the field and performing the same analysis using a different method of simulating data, and then confirming that the structure of the model does not dramatically impact the conclusions of the performance of each of the methods tested.

We have followed the useful suggestion of “stress testing” model assumptions as described in more detail in the responses to the referees. The second suggestion is less tractable at this point, as adding *var* gene diversity and tracking host memory of past infections would require an individual-based model and one that is extensively modified to become essentially very similar to our model. There are no other ABMs in the literature that we can use for this purpose without completely rewriting the model. This is because models like the Open-Malaria model (Maire N, Smith T, Ross A, Owusu-Agyei S, Dietz K, Molineaux L. A model for natural immunity to asexual blood stages of *Plasmodium falciparum* malaria in endemic areas. The American Journal of Tropical Medicine and Hygiene. 2006;75: 19–31. doi:10.4269/ajtmh.2006.75.19) include generalized immunity but not specific, adaptive, immunity of a multigene family, or consideration of such a family. Modifying such a model in a sensible way to do so would represent a major project by itself. It is not clear that this direction would provide a better model than the extensions of ours considered in the revision.

Responses to Referees' comments

We thank both referees for the constructive insightful comments which helped us clarify and improve the manuscript. In response to these comments and those of the editor, we have considered a number of extensions of the model, better explained the formulation of the model, and added to the explanation and interpretation of the results. We also note here that during the implementation of the computer code for these extensions, we realized that immigration events were not allowed from the initiation of the simulations in the original version of the work. Because this could mean that the system was not at, or close to, a stationary state, we repeated all the analyses after addressing this issue. Results remained the same as in the original submission. Also, we have now added an explicit extension that considers non-stationary dynamics and estimates MOI shortly after a temporary intervention. This provides a test of the robustness of the results during transient dynamics, more likely to represent the kinds of conditions under which the methods would be applied in the field (as referee 1 suggested).

Reviewer #1

In the submitted manuscript the authors use simulations to assess the relative performance of THE REAL MCCOIL and *var*coding in estimating the true multiplicity of infection (MOI) of a malaria infection. The *var*coding approach was previously developed by the authors in a prior publication, and THE REAL MCCOIL is a widely used approach independently developed. The issue of accurately capturing is highly pertinent for malaria control and elimination strategies, and for characterization of the epidemiology and intensity of malaria transmission. As such the authors use simulations which capture three broad levels of transmission intensity, and compare the measurement error in capturing MOI. Overall, the authors find utility in both approaches, though *var*coding provides higher accuracy especially in higher transmission settings. While the comparison is very timely, and we are in need of these tools there were a number of issues which I feel were left unresolved in the current manuscript.

We thank the referee for her/his appreciation of the work and for the constructive comments we have addressed as described below in a way that clarifies and strengthens our results.

(1) Treatment of relatedness and comparability of simulated data. As the authors state in their introduction genetically distinct malaria parasites often co-infect individuals. These co-infecting parasites may come from either a single mosquito bite, multiple mosquito bites or both. This means a single individual may be infected with closely related parasites (i.e., siblings), unrelated parasites or a spectrum of the two. In the initial THE REAL MCCOIL paper intrahost relatedness was identified as a major impact on measurement accuracy. The simulation model includes meiotic recombination, though there is no information on intrahost relatedness and how it impacts estimates of MOI from either method. How many recombinants from the initial oocyst tetrad survive to the bloodstream of the donor infection? How many oocysts per mosquito are simulated?

The referee is correct that relatedness among recombined strains from the same mosquito should influence MOI estimation. As relatedness increases, it should be more difficult to differentiate separate strains that are related.

Although our model does not explicitly represent mosquitoes, it does naturally generate strains with different levels of relatedness from meiotic recombination. Specifically, we do not model oocyst tetrads directly, but we assume that in a transmission event the number of strains that survive in the blood stage of the recipient host equals the number of strains picked up from the donor host (say N_s). The process of generating each daughter strain is as follows: 1) two parent strains are selected randomly (these could be the same strain), 2) two recombined daughter strains are generated, and one is randomly selected for transmission. This process is repeated N_s times. It mimics how the oocysts are formed and sporozoites are produced in mosquitoes and is similar to what is considered in Wong *et al.* 2018 (Wong W, Wenger EA, Hartl DL, Wirth DF. Modeling the genetic relatedness of *Plasmodium falciparum* parasites following meiotic recombination and cotransmission. PLOS Computational Biology. 2018;14: e1005923. doi:10.1371/journal.pcbi.1005923). Therefore, the relatedness of strains in our transmission model

is a natural outcome of the complex interaction between transmission intensity, MOI distribution, and host immune selection.

We now include a better description of this part of the model (L613-624) and discussion of implications for MOI estimation (L541-550). In particular, we do not expect relatedness to be an issue for our method at low-transmission, as recombination does not play an important role and MOI is sufficiently low that transmission events mostly concern donor infections with a single host. At high transmission, we also do not expect relatedness arising from recombination to be an important source of error, given the process we describe above and its realistic implementation in the model.

It appears there is a fundamental issue in the way the data is simulated. Linkage between neutral markers is considered when generating recombinants, though not (seemingly) for *var* genes where a random set of types is selected from the parents. This may have profound implications for measurement error, especially at high MOI where *var*coding shows greater benefit.

Simulations are generated with and without linkage disequilibrium between the neutral markers (S2 Table). While the association of physical locations and major groups of *var* genes is established, orthologous gene pairs between two strains are often unknown. Therefore, we implement recombination between strains as a process in which genes are randomly selected out of all the original genes from the two strains pooled together. As physical locations of *var* genes can be mobile, this assumption is a reasonable simplification of the meiotic recombination process. This clarification was added to the revised version of the manuscript (L624-630).

(2) The depth of simulations. The paper simulates three quite simplistic populations of malaria, albeit using a detailed and sophisticated model. These capture the steady state dynamics of high, medium and low transmission at the end of the wet season for seasonal malaria. While these are important categories, the utility of each approach to different settings is not explored in depth. For instance, in reality it is rare that there is instantaneous capture of parasite populations at a single time point (i.e., the end of the wet season), but

continuous capture throughout a malaria season. The MOI under the model likely increases over a season, as does the mixing among the population of parasites. Does heterogenous sampling impact measurement error (also not all malaria transmission is seasonal, does this alter error?). Notably, these approaches will be used to characterize regions where transmission has changed. This may be due to a successful intervention, a rebound post-intervention or some other scenario. How does each approach perform in the presence of recently changed transmission intensity?

The referee refers here to "simplistic" parasite populations in terms of how we sample them over time in the simulations and the assumption of stationary vs. transient dynamics. That is, the computational model is clearly complex but the settings simulated by the sampling and dynamics could be extended as suggested in the comment.

To this end, we investigated whether the sampling approach influences MOI estimation in the simulations by comparing our results to those obtained by sampling at several time-points during the malaria season. To follow field efforts in the Bongo District, a malaria-endemic area of Northern Ghana, we implemented a random sample of a total of 2000 individuals during the wet season (high-transmission season), i.e., from July (180 days) to November (300 days). These individuals were collected during five time-points (180, 210, 240, 270, and 300 days), with 400 individuals randomly sampled for each time-point. Simulations with 48 unlinked SNP loci with homogenous initial allelic frequencies for the three transmission settings were performed (S1 and S2 Tables). This experimental design results in three distinct combinations of parameters (i.e., runs) and we ran 10 replicates per combination. Our results indicated that these distinct sampling approaches did not influence the performance of both MOI estimations methods (S9 and S10 Figs). We illustrate (S9 and S10 Figs), describe (L304-306, L373-375, and L704-708), and discuss (L462-466) these new analyses in the revised version of the manuscript.

To address the second suggestion of considering samples during the transient dynamics that follow intervention, we extended our model implementation to be able to specify a reduction of transmission intensity (a temporally varying value of the contact rate) via a new parameter, "biting_rate_multiplier_by_year". This implementation can be used to apply yearlong

interventions that reduce the contact rate, the parameter specifying the temporal rate of transmission events from donor to recipient (simulating the resulting effect of a reduced biting rate or number of mosquitoes per human). In our case, a transient intervention reducing the contact rate by 50% was applied for two years immediately preceding the sampling year. We explored how this intervention influences MOI estimation by performing simulations with 48 unlinked SNP loci with homogenous initial allelic frequencies for the three transmission settings (S1 and S2 Tables). This design results in three distinct runs and we ran 10 replicates per run. As expected, the intervention reduced the prevalence of the low-transmission simulations (0.004 ± 0.003). However, consistently with the post-intervention rebound in prevalence usually observed in endemic regions (Cohen JM, Smith DL, Cotter C, Ward A, Yamey G, Sabot OJ, et al. Malaria resurgence: a systematic review and assessment of its causes. Malaria journal. 2012;11: 1–17), the intervention increased the prevalence of the moderate- and high-transmission simulations (0.491 ± 0.028 and 0.725 ± 0.012, respectively; S9 and S12 Figs). On the one hand, while the accuracy of THE REAL McCOIL approach remains similar for the moderate- and high-transmission simulations generated with or without intervention, this approach was not able to estimate MOI when an intervention was applied to the low-transmission simulations as this approach requires a minimum number of 20 hosts (S9 and S12 Figs). On the other hand, the accuracy of the *var*coding approach remains similar for simulations generated with or without interventions for the three transmission settings. As these approaches will be used to characterize regions where transmission has changed, our results support robust estimation with the *var*coding approach which does not require stationarity of the transmission dynamics. These new results are illustrated (S9 and S12 Figs), described (L308-310, L375-377, and L708-712), and discussed (L462-466) in the revised version of the manuscript.

Reviewer #2

The authors performed simulations to study the accuracy of two methods used to estimate MOI, THE REAL McCOIL and *var*coding. I think this study addresses an important

question and the results can provide insights into how to choose the most appropriate approach to estimate MOI. Since the simulations in THE REAL McCOIL paper did not show underestimation or overestimation of MOI, this manuscript can be improved by explaining this discrepancy.

We thank the referee for indicating ways in which we could clarify and extend the explanation of our findings.

1. The original THE REAL McCOIL paper presented simulation results that suggest unbiased estimates of MOI, so the readers may wonder what leads to different conclusions between studies. Is this due to the intrinsic differences between the two simulation frameworks? Can the authors provide any intuitive explanation or discuss this a bit more? I think the following analysis may help provide some hints:

As suggested by the reviewer, we provided additional explanations and discussions on the observed differences between the results based on our simulations and those presented by the original THE REAL McCOIL paper (Chang *et al.* 2017, PLOS Computational Biology). Details are outlined below.

(a) Instead of using THE REAL McCOIL to estimate allele frequencies, take the true MAF from the simulations as input, use COIL (Galinsky *et al.* 2015, Malaria Journal) to estimate MOI, and see if MOI is still over or underestimated. This will help understand whether over or underestimated MOI is due to inaccurate MAF estimates. Line 290-296 mentioned that the less accurate MOIs were explained by highly inaccurate estimated MAF - it would be helpful to explore this more systematically.

To follow this suggestion, we tried to estimate MOI based on the true minor allele frequency (MAF) from our simulations as input using COIL (Galinsky *et al.* 2015, Malaria Journal). Unfortunately, the COIL command-line version lack clear instructions, and its author Kevin Galinsky has not been able to maintain this Perl code since May 2014 and cannot provide any support as stated in one of its GitHub issues ("Web version of COIL on broadinstitute.org 504

Gateway Timeout #2”). While a web version of COIL exists, the HTML page rarely loaded properly and our analysis ran into a server problem with a message "Something went wrong", an issue which was already described in an opened and unanswered GitHub issue since May 2019 (“Server not working? #8”). Therefore, despite the interesting suggestion and considerable efforts to address it, we were unable unfortunately to explore the relation between MOI and the MAF accuracies with this more systematic approach.

(b) After controlling for MAF (using the true MAF), if MOI is still over or underestimated, it is likely because the frequency of DAC is different from that expected in the model implemented in THE REAL McCOIL (Equation 2). If the proportion of DAC is higher (or lower) than expected from the REAL McCOIL model, MOI is likely to be overestimated (or underestimated). If this is the case, it would be helpful if the authors could provide some explanation for this (for example, can diversifying selection or immune selection and linkage between neutral and non-neutral markers explain why individuals with high MOI show lower than expected diversity and individuals with low MOI show higher than expected diversity)?

When only keeping simulations with the lowest estimated MAF inaccuracy per simulation (inaccuracy < 0.39 = 1st quantile), defined as the sum of the absolute differences between estimated and true MAF per SNP locus, THE REAL McCOIL still tends to overestimate MOI when true values range from 3 to 14 and to underestimate MOI for true MOI above ~14 (S14 Fig). This observation suggests that the estimated MAF inaccuracy is not the only source of inaccuracy of the estimated MOI. For these simulations with a more accurate MAF, the hosts with overestimated MOI have a higher proportion of DACs (82.4% ± 17.8%) than those for which MOI was underestimated (48.1% ± 34.8%), which is consistent with the proportion of SNP calls (genotypes) per host SNP haplotype (Fig 3). Therefore, hosts with high MOI showed lower diversity than expected in the model implemented in THE REAL McCOIL (Equation 2). These new results are illustrated (S14 Fig) and described (L338-342) in the revised version of the manuscript.

2. Line 238-239, “Interestingly, while most hosts with MOI < 3 show accurate MOI estimates, some can differ from the true MOI by up to 18, 12, and 13 co-infections for the

low-, moderate-, and high-transmission simulations, respectively." Do these individuals show unusually high proportions of DAC?

Indeed, as described in the Results section of the manuscript (L260-263), the inaccuracy of the MOI estimates based on THE REAL McCOIL approach, defined as the absolute differences between estimated and true MOI per host, is significantly and positively correlated with the proportion of DACs per host. While this result already indicates that individuals having the least accurate MOI estimates show unusually high proportions of DACs, we also compared the proportion of each type of call of these individuals to help better understand the source of this inaccuracy. Our results show that these hosts correspond to those with a true MOI of 1 (i.e., proportion of DACs = 0%, Fig 1 and Fig 3, which belong to nine low-transmission simulations, two replicates of runs 12 and one replicate of runs 6, 10, 15, 18, 20, 21, and 22; Fig 1A; S2 Table). These hosts showed a high proportion of missing calls (e.g. 37.7% ± 12.0% when the MOI estimates differ from the true MOI by 13 co-infections) and/or a higher proportion of minor single calls (e.g. 54.7% ± 2.5% when the MOI estimates differ from the true MOI by 18 co-infections) than major single calls (e.g. 32.9% ± 7.4% when the MOI estimates differ from the true MOI by 18 co-infections), which may thus contribute to highly inaccuracy estimates despite the absence of DACs. These new results are described (L316-325) in the revised version of the manuscript.

3. The 95% credible interval of MOI from THE REAL McCOIL may be wider for higher values of true MOI. Therefore, I suggest also using "whether the 95% credible interval of MOI contains true MOI" to assess the accuracy of MOI estimates.

As recommended, we compared the true MOI with THE REAL McCOIL 95% MOI credible interval. Interestingly, a high proportion of the 95% credible intervals contained the true MOI for the distinct transmission settings (86% ± 35%, 96% ± 21%, and 97% ± 18% for the low-, moderate-, and high-transmission simulations, respectively). However, as the referee suggests, the length of this 95% credible interval is significantly and positively correlated with the true MOI (r = 0.80, *P*-value < 2.2e-16), increases with transmission intensity (0.3 ± 1.6, 0.7 ± 2.2, and 3.6 ± 5.2 for the low-, moderate-, and high-transmission simulations, respectively; S4 Fig), and can reach up to 19, 18, and 17 co-infections for the low-, moderate-, and high-transmission simulations, respectively.

We illustrated (S4 Fig) and described (L252-258) these results in the revised version of the manuscript.

Minor comments

1. Does Fig 3 present values from simulations for high-, moderate-, or low-transmission setting? I noticed that the proportion of DAC is 0 for true MOI = 1, but in Fig 1A, the estimated MOI can be greater than one low transmission setting, which is confusing.

Indeed, Fig 3 presents values from simulations for low-, moderate-, and high-transmission settings. To avoid potential confusion, we added this information in the caption of Fig 3 (L281-282) and provided an additional figure illustrating the proportion of SNP calls per host for each transmission intensity (S3 Fig). As monoclonal infections (i.e., MOI = 1) only contain one allele per locus the double allele count (DAC) is always 0 for these infections, i.e., each locus only presents one single allele (0 or 1). As explained in our answer to the second comment of the second reviewer, estimated MOI can be greater than one for hosts with a true MOI of 1 from nine low-transmission setting simulations (i.e., two replicates of runs 12 and one replicate of runs 6, 10, 15, 18, 20, 21, and 22; Fig 1A; S2 Table). These hosts showed a high proportion of missing calls (e.g. 37.7% ± 12.0% when the MOI estimates differ from the true MOI by 13 co-infections) and/or a higher proportion of minor single calls (e.g. 54.7% ± 2.5% when the MOI estimates differ from the true MOI by 18 co-infections) than major single calls (e.g. 32.9% ± 7.4% when the MOI estimates differ from the true MOI by 18 co-infections), which may thus contribute to highly inaccurate estimates despite the absence of DACs. These clarifications were added to the revised version of the manuscript (L316-325).

2. Line 586-588 "We performed the categorical method of THE REAL McCOIL with a minor allele frequency (MAF) of 10% for a SNP to be considered." Why were only SNPs with MAF >10% considered? Are the results similar when another threshold (such as 5% or 1%) is used? For example, line 281-284, "Consequently, due to the high number of SNP loci, and thus individuals, that could not be considered in THE REAL McCOIL analysis, MOI could not have been estimated for any of the low-transmission setting simulations when a

subsampling of 200 individuals was applied (S6 Fig)." Can MOI be estimated if the threshold is lowered?

Indeed, MOI can be estimated if the minor allele frequency (MAF) threshold is lowered. However, it is generally recommended that the SNPs have a MAF greater than 10%. While a MAF ≥ 10% indicates that the locus can sufficiently discriminate between isolates in the population, a MAF < 10% indicates that a locus is not representative, that alleles were moving towards fixation in the population, and that this locus would not be informative to differentiate isolates from each other in the population. Moreover, Daniels *et al.* 2008 used a higher MAF cutoff than ≥ 10% during the development of their 24-SNP barcode (i.e., "SNP exhibiting an average MAF of at least 35% … were chosen for final assay development"; Daniels R, Volkman SK, Milner DA, Mahesh N, Neafsey DE, Park DJ, et al. A general SNP-based molecular barcode for *Plasmodium falciparum* identification and tracking. Malar J. 2008;7: 223. doi:10.1186/1475-2875-7-223). Therefore, as MAFs impact the inference of population structure, these loci were removed from downstream analyses. This clarification was added to the revised version of the manuscript (L679-682).

3. How "distinct initial SNP allele frequencies" are set (were these frequencies sampled from a particular distribution)? It seems that it makes more sense to consider various allele frequencies for the default setting (compared to a constant frequency). Also, I only saw a range for the constant frequency in Table S1, but could not find what value was used.

For each locus, the migrant alleles from the regional pool (0 or 1) were sampled with distinct initial probabilities summing up to one (e.g. 0.2 and 0.8). These probabilities were randomly and uniformly picked from a defined range. To avoid simulations potentially containing several fixed, and thus useless, SNP loci (see our answer for the previous minor comment), we simulated SNP with an initial allele frequency from a range only allowing frequencies greater than 10% or lower than 90% (S1 Table). However, while all loci have an initial MAF ≥ 10%, their SNP allele frequencies can change over time as a function of the transmission dynamics of *P. falciparum*. These clarifications were added to the revised version of the manuscript (L652-656 and L679-682).

4. If I understand it correctly, the true MOI defined in this study is the number of coinfections, no matter whether the parasites in different infections are genetically similar or not. However, in the abstract, MOI is defined as the number of genetically distinct parasite strains, and how to define "genetically distinct" is usually unclear and can lead to confusion. It would be helpful to explain/define these more clearly in the manuscript. For example, if an individual is infected twice and the parasites are 50% similar, is true MOI 2 or 1.5?

Very useful suggestion. Indeed, the true MOI defined in this study corresponds to the number of coinfections, no matter whether the parasite in the different infections are genetically similar or not. This clarification was added to the revised version of the manuscript (L192-194).

5. Line 344-346, "Consistent with the average PTS, the similarity networks for simulations under <u>high</u>-transmission settings did not group the *var* repertoires into well-defined modules while the similarity networks for simulations under <u>high</u>-transmission settings did." There seems to be a typo here; one of them should be low- and moderate-.

Indeed, there was a typo here. We corrected this sentence as follow: "Consistent with the average PTS, the similarity networks for simulations under **high**-transmission settings did not group the *var* repertoires into well-defined modules while the similarity networks for simulations under **low**- and **moderate**-transmission settings did" (L386-388).

6. Line 435-437, "Surprisingly, although THE REAL McCOIL approach assumes that genotyped SNP loci do not exhibit significant LD, the simulations performed with linked SNP loci did not show less reliable MOI estimation." The same finding was also reported in the original paper.

Thank you for indicating this. We replaced this sentence with the following one: "Consistently with Chang *et al.* 2017, although THE REAL McCOIL approach assumes that genotyped SNP loci do not exhibit significant LD, the simulations performed with linked SNP loci did not show less reliable MOI estimation." (L483-485).

7. Line 591-593, "… a probability of calling single allele loci double allele loci and of calling double allele loci single allele loci of 0.05 which were estimated with MOI and the allele frequencies) [21,66]". I suggest adding "initial" to make it clearer. "… an initial probability of calling single allele loci double allele loci and of calling double allele loci single allele loci of 0.05 which were estimated with MOI and the allele frequencies) [21,66]".

Following the suggestion, we replaced this sentence with the following one: "… an initial probability of calling single allele loci double allele loci and of calling double allele loci single allele loci of 0.05 which were estimated with MOI and the allele frequencies) [21,66]" (L677-679).

Have the authors made all data and (if applicable) computational code underlying the findings in their manuscript fully available?

The PLOS Data policy requires authors to make all data and code underlying the findings described in their manuscript fully available without restriction, with rare exception (please refer to the Data Availability Statement in the manuscript PDF file). The data and code should be provided as part of the manuscript or its supporting information, or deposited to a public repository. For example, in addition to summary statistics, the data points behind means, medians and variance measures should be available. If there are restrictions on publicly sharing data or code —e.g. participant privacy or use of data from a third party— those must be specified.

Reviewer #1: Yes

Reviewer #2: None

The SNP data used for this analysis are available in Dryad at https://doi.org/10.5061/dryad.jsxksn0bp. The DBLα sequences used for this analysis are available in GenBank under BioProject Number: PRJNA 396962. The code will be made available at https://github.com/pascualgroup varmodel3 upon acceptance.

PLOS authors have the option to publish the peer review history of their article (what does this mean?). If published, this will include your full peer review and any attached files.

If you choose "no", your identity will remain anonymous but your review may still be made public.

Do you want your identity to be public for this peer review?
For information about this choice, including consent withdrawal, please see our Privacy Policy.

Reviewer #1: No

Reviewer #2: No

Figure Files:

While revising your submission, please upload your figure files to the Preflight Analysis and Conversion Engine (PACE) digital diagnostic tool, https://pacev2.apexcovantage.com. PACE helps ensure that figures meet PLOS requirements. To use PACE, you must first register as a user. Then, login and navigate to the UPLOAD tab, where you will find detailed instructions on how to use the tool. If you encounter any issues or have any questions when using PACE, please email us at figures@plos.org.

Data Requirements:

Please note that, as a condition of publication, PLOS' data policy requires that you make available all data used to draw the conclusions outlined in your manuscript. Data must be deposited in an appropriate repository, included within the body of the manuscript, or uploaded as supporting information. This includes all numerical values that were used to generate graphs, histograms etc.. For an example in PLOS Biology see here:

http://www.plosbiology.org/article/info%3Adoi%2F10.1371%2Fjournal.pbio.1001908#s5.

Reproducibility:

To enhance the reproducibility of your results, we recommend that you deposit your laboratory protocols in protocols.io, where a protocol can be assigned its own identifier (DOI) such that it can be cited independently in the future. Additionally, PLOS ONE offers an option to publish peer-reviewed clinical study protocols. Read more information on sharing protocols at https://plos.org/protocols?utm_medium=editorial-email&utm_source=authorletters&utm_campaign=protocols
